# The ABC of Heart Transplantation—Part 1: Indication, Eligibility, Donor Selection, and Surgical Technique

**DOI:** 10.3390/jcm12165217

**Published:** 2023-08-10

**Authors:** Daniele Masarone, Michelle M. Kittleson, Luigi Falco, Maria L. Martucci, Dario Catapano, Benedetta Brescia, Andrea Petraio, Marisa De Feo, Giuseppe Pacileo

**Affiliations:** 1 Heart Failure Unit, Department of Cardiology, AORN dei Colli-Monaldi Hospital, 80131 Naples, Italy; 2 Department of Cardiology, Smidt Heart Institute, Cedars-Sinai Hospital, Los Angeles, CA 90048, USA; 3 Department of Experimental Medicine, University of Campania “Luigi Vanvitelli”, 80131 Naples, Italy; 4 Heart Transplant Unit, Department of Cardiac Surgery and Transplants, AORN dei Colli Monaldi Hospital, 80131 Naples, Italy; 5 Cardiac Surgery Unit, Department of Cardiac Surgery and Transplants, AORN dei Colli Monaldi Hospital, 80131 Naples, Italy

**Keywords:** advanced heart failure, heart transplant, patient referral, patient selection

## Abstract

Cardiac transplantation represents the gold standard of treatment for selected patients with advanced heart failure who have poor functional capacity and prognosis despite guideline-directed medical therapy and device-based therapy. Proper patient selection and appropriate referral of patients to centers for the treatment of advanced heart failure are the first but decisive steps for screening patients eligible for cardiac transplantation. The eligibility and the decision to list for cardiac transplantation, even for patients with relative contraindications, are based on a multidisciplinary evaluation of a transplant team. This review will discuss the practical indications, the process of patient eligibility for cardiac transplantation, the principle of donor selection, as well as the surgical technique.

## 1. Introduction

Since the first transplant performed in humans by Christiaan Barnard in Cape Town in 1967 [1], heart transplantation (HT) has become an established therapy for patients with heart failure (HF), representing, to date, the gold-standard treatment for end-stage HF [2].

Due to limited donor supply, a decline in the number of HTs was observed between the 1990s and early 2000s; however, in recent years, the number of HT patients has increased, and about 5500 HTs have been performed annually worldwide in recent years [3].

Because the management of the patient candidate for or with cardiac transplantation requires specific skills that not all general internists and cardiologists possess, to provide practical clinical practice advice for managing a progressively growing problem, we decided to write a pragmatic guide to cardiac transplantation divided into two parts. The first part will discuss indications, contraindications, donor selection, and surgical techniques, while the second part will cover postoperative care, the principle of immunosuppression, and short- and long-term complications after HT.

## 2. Referral of the Patients for HT

**Figure 1:**
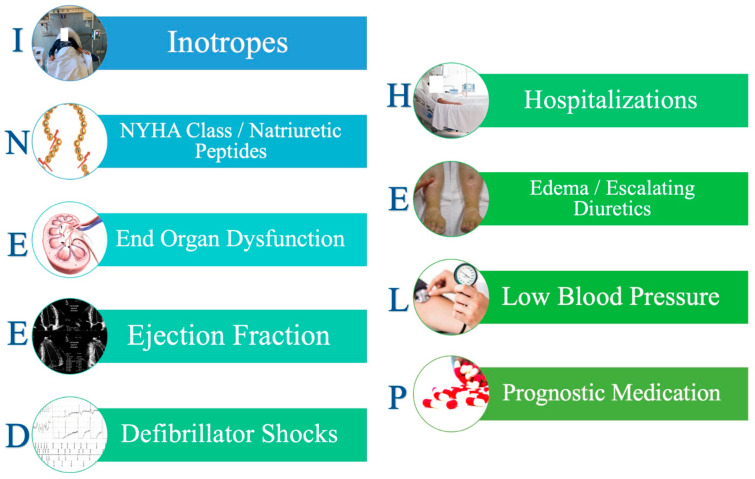
I NEED HELP, a simple mnemonic to remember the “red flags” for the patient’s referral to an advanced heart failure center. I: Previous or ongoing requirement of inotropes or inodilators. N: NYHA class III-IV, high natriuretic plasma levels, E: worsening kidney or liver function due to heart failure, E: left ventricular ejection fraction <20%, D: recurrent appropriate defibrillator shocks, H: 2 or more episodes of worsening heart failure, E: persisting fluid overload and/or increasing diuretic requirement, L: systolic blood pressure persistently <90–100 mm Hg, P: intolerance to disease-modifying drugs.

Early identification of the patient as a candidate for HT is the first step in proper patient selection. To this end, those clinical elements that characterize an abysmal prognosis, such as poor hemodynamic status, frequent episodes of worsening heart failure, intolerance to guideline-directed medical therapy, and worsening renal and hepatic function, should be carefully sought at every visit of patients with HF [4]. An intuitive acronym for remembering these elements is “I NEED HELP” [5] (Figure 1). The presence of even one of these warning elements indicates the need for a referral of the patient to an advanced HF center to evaluate the possible indication for HT [6].

## 3. Indications to HT

HT represents the gold standard of treatment for selected patients with advanced HF who have poor functional capacity and prognosis despite guideline-directed medical therapy and device-based therapy. It is generally considered when it is likely to increase survival and improve quality of life [7]. However, due to the limited number of available hearts, only a few eligible patients can receive HT. The ongoing organ donor shortage limits the number of HF in which HT can be performed. Therefore, carefully selecting patients suitable for HT is critical to ensure the best use of the limited organ available. As shown in Table 1, except for the International Society for Heart and Lung Transplantation (ISHLT) recommendations [8], other international guidelines [9,10] are vague regarding the indication for HT; therefore, we provide a practical approach for the appropriate selection of transplant candidates. The class of recommendation (COR) and the level of evidence (LOE) are provided according to the last guidelines of ISHLT [5].

### 3.1. Cardiopulmonary Exercise Test

Peak VO_2_ is the main parameter to consider in outpatients with advanced HF as it is closely related to the prognosis of such patients [11]. It is defined by the Fick equation as the product of cardiac output (stroke volume × heart rate) and arteriovenous oxygen difference [C (a–v) O_2_] at peak exercise, and it establishes the limits of the cardiopulmonary system [12].

In a milestone study including 114 outpatients with advanced heart failure referred for HT, Mancini et al. showed that patients with a VO_2_ peak < 14 mL/kg/min had significantly lower one-year survival than patients with a VO_2_ peak > 14 mL/kg/min^2^ (95% vs. 70%; *p* < 0.005) [13].

Accordingly, the latest criteria published by the ISHLT to guide inclusion in the HT proposed two different VO_2_ peak cut-offs depending on whether the patient is (peak VO_2_ ≤ 14 mL/kg/min) or not (peak VO_2_ ≤ 12 mL/kg/min) on β-blocker treatment (COR I, LOE B) [5].

Although a specific value of peak VO_2_ is not clearly stated in the latest European Society of Cardiology guidelines on the diagnosis and treatment of HF, it is worth mentioning that the Heart Failure Society Association of the European Society of Cardiology’s definition of advanced HF, severe limitation of functional capacity is indicated as low 6 MWT distance (<300 m) or pVO2 < 12 mL/kg/min or <50% predicted value [14].

Therefore, the main indication for HT is a peak VO_2_ value < 12 mL/kg/min (COR I, LOE B) or, in outpatients aged < 50 years, a peak VO2 value < 50% of the expected value (COR Iia, LOE B).

The slope of the relationship between ventilation (VE) and arterial carbon monoxide volume (VCO_2_) describes the patient’s ventilatory efficiency (i.e., the amount of air released during exercise) [15].

The VE/VCO_2_ slope represents another significant predictor of mortality and cardiovascular events in patients with advanced HF.

In a study enrolling 213 advanced HF patients who were candidates for HT, Arena et al. demonstrated that patients with a VE/VCO_2_ slope < 34 had better survival than patients with a VE/VCO_2_ value > 34 (99% vs. 83%; *p* < 0.005) [16].

Based on these data, ISHLT guidelines indicate the use of a VE/VCO_2_ slope of >35 as a determinant in listing for OHT in the presence of a submaximal cardiopulmonary exercise test (RER < 1.05); however, it should be stressed that this indication is based only on experts’ opinion (COR IIb, LOE C).

However, placing a patient on the waiting list for HT based only on cardiopulmonary testing is not recommended; therefore, the patient’s functional capacity, as well as heart failure prognostic scores, should also be evaluated for listing patients for HT.

### 3.2. Heart Failure Prognostic Scores

The most widely used risk scores in patients with advanced HF are the Seattle Heart Failure Model (SHFM) and the Heart Failure Survival Score (HFSS) [17]. Survivorship of less than 80 percent at one year, estimated by the SHFM, is generally considered a reasonable cut-off for listing for HT (COR IIb, LOE C). However, it should be regarded that the SHFM tends to overestimate survival and has extremely limited sensitivity in younger patients with advanced HF [18]. In contrast, a low risk predicted by HFSS (score ≥ 8.1, 1-year survival 93%) generally rules out the need for HT (COR IIb, LOE C).

## 4. Contraindications to HT

Table 2 summarizes the various conditions that represent “absolute” contraindications to HT. These conditions are generally relative and not absolute and should be evaluated based on the patient clinical profile, the potential benefits of HT, and the availability.

### 4.1. Age

The number of patients aged >60 years undergoing HT has increased over the past 10 years, as 5-year survival in carefully selected patients aged >70 years is estimated to be about 70% [19].

In addition, a recent retrospective analysis of the Scientific Registry of Transplant Recipients showed that transplant recipients aged ≥70 years had poorer survival than younger recipients (log-rank *p* = 0.03). However, after adjustment for risk predictors in the Cox proportional hazards model (body mass index, serum creatinine, total score at Index for Mortality Prediction After Cardiac Transplantation), no significant difference in the 5-year mortality between older and younger recipients (HR 1.06, 95% CI 0.91–1.254; *p* = 0.43) was found [20].

Despite these data, given the scarcity of donors and the recently demonstrated 5-year survival for the last-generation device for left ventricular assistance (Heartmate 3), we recommend limiting HT in patients >70 years of age in highly selected patients and for all others to perform Heartmate 3 implantation as destination therapy.

### 4.2. Pulmonary Hypertension

The combination of systolic pulmonary pressure >50 mm Hg, transpulmonary gradient > 15 mm Hg, and pulmonary vascular resistances (PVR) > 3 Wood Unit not reversible with a vasodilator challenge is an absolute contraindication to the listing for HT (COR I, LOE C) [21]. This recommendation is due to the risk of acute right HF due to an insufficient adaptation of the donor’s heart to high PVR [22]. Therefore, in such patients, a prolonged (usually up to 48–72 h) attempt at the reversibility of pulmonary vascular resistance with in-hospital use of diuretics, inotropes, and vasodilators should be made before definitive exclusion from the transplant list (COR I, LOE C) [23]. As a last resort, ventricular unloading with left-ventricular-assist device implantation as a bridge to transplantation may be considered (COR IIib, LOE C), particularly in young patients (age < 60 years) who have no other contraindications to HT [24].

### 4.3. Kidney Dysfunction

Kidney dysfunction (KD) is caused by several factors in patients with advanced HF; if it is due to low cardiac output [25], renal venous congestion [26], or both (functional kidney disease), it may reverse after appropriate pre-HT management (inotropes/vasodilators/diuretics, temporary renal replacement therapy) and is not a contraindication to HT [27,28]. Otherwise, intrinsic (structural) KD may represent a contraindication to HT [29]. Therefore, in patients with KD who should be candidates for HT, it is necessary to identify through a multiparametric evaluation (Table 3) those forms of KD that should improve with the hemodynamic optimization offered by HT [30]. A retrospective review of data from the 30,090 patients undergoing HT included in the UNOS database showed that prognosis is closely influenced by the estimated glomerular filtration rate (eGFR); e-GFR before HT was predictive of end-stage renal disease after HT and the need for a kidney transplant, with the highest risk in subjects with pre-OHT eGFR < 30 mL/min/1.73 m^2^ (adjusted Hazard Ratio 1.55 (95% CI 1.41–1.70)) [31].

According to these data, international guidelines consider an eGFR < 40 mL/min/m^2^ a relative contraindication to HT (COR IIa, LOE C); in these patients, a combined heart–kidney transplant should be considered if KD is considered related to intrinsic disease or if eGFR is <30 mL/min/m^2^ [32]. Otherwise, patients with eGFR > 40 mL/kg/min do not need combined heart–kidney transplantation.

It should be underscored that these recommendations are also based on the consideration that immunosuppressive regimens used after HT (particularly calcineurin inhibitors) may further worsen renal function [33].

### 4.4. Liver Dysfunction

Liver dysfunction in patients with advanced HF may be due to congestive hepatopathy [34], with or without fibrosis (which may be reversible), liver hypoperfusion with or without hepatocellular necrosis (which is irreversible) [35], or other coexisting liver diseases [36].

Because the accurate assessment of liver function is often complex in patients with advanced HF (increased transaminase values only in advanced stages of the disease, possibility of diagnosing liver fibrosis only by transient elastography and not by standard ultrasonography), several risk scores have been tested for estimating the risk of liver complications and mortality before HT [37].

The Model for end-stage liver disease excluding the INR (MELD-XI) score is the most used and strongly predictive of prognosis after OHT. Indeed, in a retrospective analysis of 617 patients undergoing HT, patients with a MELD-XI score < 14 had a 5-year survival rate of 82.2% compared with 55.1% in those with a MELDXI score > 20 [38].

The recent recommendations from the American Heart Association suggest a hepatology consultation in patients with clinical–instrumental markers of advanced liver disease or for possible liver disease to consider the need for a combined heart–liver transplant (Table 4) [30].

### 4.5. Diabetes Mellitus

Type 2 diabetes mellitus (T2DM) is a common comorbidity in patients with advanced HF. It is often associated with several extracardiac conditions [39] that can complicate the post-HT course, such as obesity, increased risk of infection, renal dysfunction, and transplant coronary artery disease [40].

In a recent single-center study, Rivinius et al. showed that patients with T2DM and HbA1c < 7.0% have a significantly better 5-year post-HT survival than patients with HbA1c ≥ 7.0% (68.7% vs. 46.3%; *p* = 0.008) [41]. These results emphasize the clinical relevance of a well-controlled T2DM in OHT recipients. Considering these results and the fact that the immunosuppressive regimen (particularly corticosteroid) can worsen glycemic control the ISHLT guidelines state that diabetes mellitus with end-stage organ damage (other than non-proliferative retinopathy) or persistent poor glycemic control (i.e., HbA1c > 7.5% or 58 mmol/mol), despite optimal pharmacological therapy, is a relative contraindication to OHT (cOR IIa, LOE C).

### 4.6. Obesity

In recent years, much evidence has demonstrated the negative effect of a high body mass index (BMI) value on outcomes after HT [42]. A BMI value between 30 and 35 kg/m^2^ is not associated with increased mortality after HT [43]; conversely, patients with a BMI > 35 kg/m^2^ have not only longer waiting times and a lower probability of finding a suitable donor but also increased post-transplant morbidity and mortality [44]. Therefore, the ISHLT recommends attaining a BMI < 35 kg/m^2^ before listing an obese patient for HT (COR IIa, LOE C).

### 4.7. Cancer

Improved survival of cancer patients has led to an increase in the cohort of patients with a history of malignancy undergoing HT in recent years [45]. For these patients, the risk of recurrence or de novo cancer has increased about 2.5–4-times compared with the general population of the same age [46,47]. Clinically, it should be considered that pre-existing neoplasms are different with a different risk or recurrence [48]. Therefore, a multidisciplinary cardio-oncology team should perform the pre-transplant evaluation of a patient with previous malignancy by carefully assessing the risk of recurrence and listing for HT patients with a low risk (Class I, Level of evidence C). As a general rule and according to a recent consensus expert opinion statement [49], a disease-free interval of 5 years is required for malignancies at high risk of recurrence (e.g., colorectal cancer, hormone receptor-negative breast cancer) and an interval of 3 years for cancer at intermediate risk of recurrence (e.g., prostate cancer, renal cancer, bladder cancer).

## 5. Donor Selection

Donor selection for HT involves identifying and evaluating patients potentially eligible for heart donation [50].

### 5.1. Identification of Potential Organ Donors

In general, potential organ donors are patients mechanically ventilated in a state of brain death (donation after brain death (DBD)) or in a state of circulatory death (donation after circulatory death (DCD)). To date, the majority of donations are DBDs, while only a minority are DCDs [51].

Brain death is declared when all brain function has ceased. Planned organ procurement in DBD is a well-established process that involves the cessation of organ function by flushing with a cold solution. On the other hand, DCD donors do not meet the criteria for complete brain death but have devastating and irreversible injuries that make continued care futile and unnecessary. After the withdrawal of vital support, circulatory death is declared based on the absence of blood pressure and heartbeat for a period of at least five minutes. Historically, hearts with DCD suffer severe ischemic damage that makes their use for HT impossible; however, in recent years, improvements in artificial perfusion (particularly ex situ perfusion via TransMedics Organ Care System) allow for resuscitation of DCD hearts, making their use for HT possible [52,53]. According to some recent estimates, the use of such artificial perfusion techniques can increase the donor pool by up to 50%.

### 5.2. Evaluation of Potential Donors

The first stage in evaluating potential donors for HT includes confirmation of brain death or establishment of circulatory death, as well as verification of consent to the donation by the parents. Once brain or circulatory death and willingness to donate have been confirmed, several demographic, clinical, and instrumental characteristics are considered to confirm eligibility for donation. The main factors that are considered to confirm donors’ suitability will be discussed below. The CORs and LOEs are provided according to recently published evidence-based guidelines for providers [54].

#### 5.2.1. Age

- Age < 45 years is recommended for heart donors (COR I, LOE C).
- Donors older than 45 years may be considered after careful screening for the presence of significant coronary artery disease (e.g., narrowing ≤ 50%) and if graft ischemia times <4 h is expected (COR I, LOE C).
- Older donors can be considered for older recipients or highly sensitized recipients with negative crossmatch (COR IIa, LOE C).

#### 5.2.2. Size

- Assignment of hearts from female donors to male recipients can be performed safely, especially in the absence of pulmonary hypertension or when the predicted heart mass value in the recipient is within 20% to 30% (COR I; LOE C).

#### 5.2.3. Hemodynamic Status

- Hemodynamic support with low doses of norepinephrine (e.g., ≤0.1 μg/kg/min) is compatible with donor suitability if other (COR I; LOE C).
- If a high dose of norepinephrine or use of combined inotropes/vasopressor is necessary to maintain adequate circulatory function in the donor, the use of a Swan-Ganz catheter is recommended to verify the radius achievement of hemodynamic goals (mean arterial pressure > 60 mm Hg, cardiac index > 2.4 L/min/m^2^, central venous pressure < 12 mm Hg, pulmonary capillary wedge pressure < 12 mm Hg; COR IIa, LOE C).

#### 5.2.4. Metabolism Status

- Moderately abnormal serum sodium is not a criterion of exclusion of the donor; conversely, hearts from donors with extreme hyponatremia (serum sodium < 129 mEq/L) or hypernatremia (serum sodium > 170 mEq/L) should not be used for HT (COR IIa, LOE C).
- Donor diabetes is not a criterion of exclusion of the donor (COR IIa, LOE C); however, coronary angiography should be performed in all diabetic donors (COR IIa, LOE C).

#### 5.2.5. Systolic Function

Hearts with systolic dysfunction documented by echocardiography, especially from young donors in a brain-dead state, should not be discarded for donating; in fact, it is reasonable to repeat echocardiographic evaluations to determine improvements in the heart of such donors.

#### 5.2.6. Blood Group and Anti-Human Leukocyte Antigen (HLA) Compatibility

- ABO blood group compatibility between donor and recipient should be confirmed (COR I, LOE C).
- Preformed human leukocyte antigen (HLA) antibodies should be researched in the recipient and compared against the donor HLA, at least virtually (COR IIa, LOE C).

#### 5.2.7. Comorbidities

- Left ventricular hypertrophy should be detected by echocardiography in all donors (COR I, LOE C).
- Coronary artery disease should be researched by coronary angiography in patients with (COR IIa, LOE C):
  - Age > 45 years
  - Diabetes
  - Hypertension
  - Obesity
  - Hyperlipidemia
  - Tobacco and/or cocaine/methamphetamine use
- Carefully selected donor hearts with an interventricular septum and/or posterior wall thickness *≥* 13 may be suitable for HT, particularly with donors ≤ 40 years of age (COR IIa, LOE C).
- Heart from donors with diabetes, chronic hypertension as well as coronary artery disease with mild luminal irregularities (e.g., ≤50% narrowing) can be safely used (COR IIa, LOE C).

#### 5.2.8. Drug Use

- The heart of donors with a history of tobacco, cocaine, and amphetamine abuse can be safely used for HT if the systolic function is normal, maximal wall thickness is < 14 mm, and coronary angiography is normal (COR IIa, LOE C).

## 6. Surgical Technique

**Figure 2:**
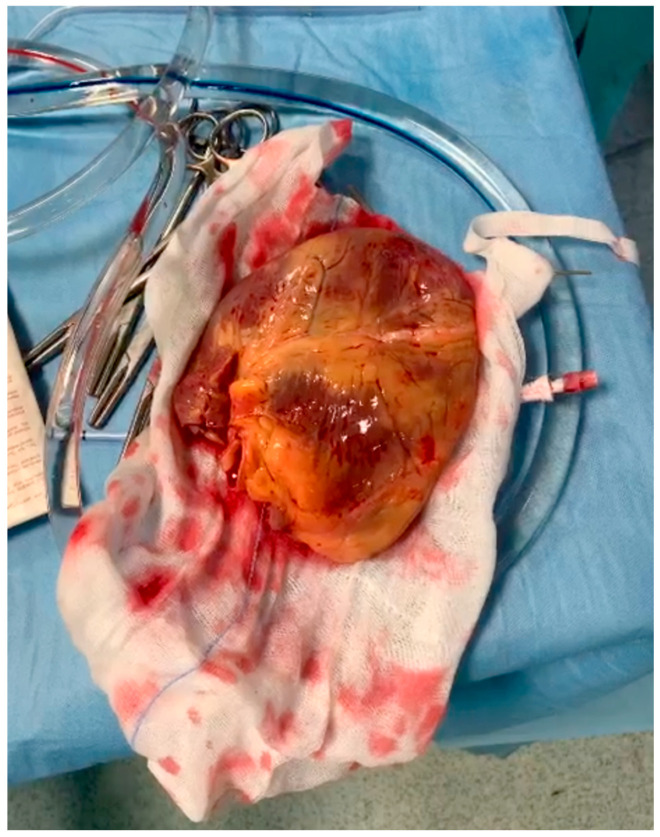
A donor’s heart waiting to be transplanted into the recipient.

Once a suitable heart is available, the recipient is contacted by telephone for admission to the cardiac surgical setting. Assessing immunologic and blood group compatibility is essential at this stage as evaluating the patient’s coagulation status (platelet count and prothrombin time) and new or ongoing infections in the recipient. Generally, careful planning of the entire HT is attempted to limit the donor ischemia time to less than 4 h [55]. Once the donor’s heart arrives (Figure 2), HT is performed, which consists of several consecutive steps.

### 6.1. Initial Setup

The preparation of the recipient involves an initial phase, like all open-heart surgery. After a median sternotomy, a cardiopulmonary bypass is made via cannulation of the aorta and great veins (in both cases, cannulation is performed as distally as possible to facilitate the subsequent stages of HT).

### 6.2. Recipient Cardiectomy

After placing the patients in cardiopulmonary bypass, the aorta is clamped, and the caval snares are tightened. Next, the aorta is separated from the pulmonary artery (distally to the valve structures), and the interatrial groove is incised. The superior vena cava is transected at the cavo-atrial junction, while a wide inferior vena cava cuff is made.

Finally, through the left atrial roof, an incision is made over the left atrial cuff to leave an adequate portion of posterior left atrial tissue. In contrast, the left atrial appendage is usually excised.

### 6.3. Preparation of Donor’s Heart

As a first operation, the large arterial vessels (aorta and pulmonary arteries) are separated, and the attachments of the posterior pulmonary artery to the left atrium are incised. The left atrial cuff is prepared by connecting the incisions between the four pulmonary veins.

If the bicaval technique is chosen, the superior vena cava cuff is cut at the level of the azygous vein opening. The bicaval approach is preferred due to the better preservation of the geometry of the right atrium and tricuspid valve [56,57]. However, if the biatrial technique is used, the superior vena cava is ligated, and the right atrium is opened from the inferior vena cava to the right atrial appendage.

Any foramen ovale of the recipient’s heart is also searched and closed at this stage.

### 6.4. Creation of Anastomosis

Generally, anastomoses are performed in the following order: left atrium, pulmonary artery, aorta, inferior vena cava, and superior vena cava. If necessary, as in the case of hearts with ischemia time >4 h or “marginal” donor hearts, the aortic cross-clamp may be removed immediately after left atrial and aortic anastomosis to allow for earlier graft reperfusion. However, the aortic cross-clamp is commonly removed after all anastomoses are completed.

Anastomoses are performed with double-armed running 3-0 polypropylene sutures for the left atrium and a double-armed 4-0 polypropylene suture for the pulmonary artery, aorta, and superior and inferior vena cava.

### 6.5. Final Setup

After completing all anastomoses, if not performed previously, one removes the aortic cross-clamp and waits sufficient time for reperfusion. During this period, suture lines are checked for proper execution, temporary atrial and right ventricular pacing wires are placed, and standard chest tubes are placed. The patient is then sent to the intensive care unit for the continuation of care.

## 7. Conclusions

Although more than 50 years have passed since the first HT in 1967, this therapeutic option remains virtually unknown to most of the cardiology community. The key to successful heart transplantation is the appropriate and timely selection of the patient to be a candidate for transplantation because, although short- and long-term survival has improved, the limited donor pool limits the number of patients who can access this option. Therefore, it is the moral obligation of all those working in this field to have a clear understanding of the principles governing the selection of patients to be candidates for HT for the potential benefit of each donated organ.

## Figures and Tables

**Table 1:** Indications to heart transplantation according to international guidelines. HF: Heart Failure, CPET: Cardiopulmonary Exercise Test, SHFM: Seattle Heart Failure Model, HFSS: Heart Failure Survival Score, GDMT: Guidelines Directed Medical Therapy.

ISHLT	ACC/AHA	ESC
Advanced HF is defined according to CPET parameters and Prognostic HF score. Maximal CPET: VO_2_ peak ≤ 12 mL/kg/min (≤ 14 if patients intolerant to β-blockers) or VO_2_ peak < 50% of the predicted value in patients with age < 50 years Submaximal CPET: VE/VCO_2_ slope ≥ 35 Survival at SHFM: ≤80% Range at HFSS: High risk	Advanced HF despite GDMT	Advanced HF, refractory to medical/device therapy, and without absolute contraindications.

**Table 2:** Absolute and relative contraindications to heart transplantation. PAS: Pulmonary Artery Systolic Pressure, TPG: Trans Pulmonary Gradient, PVR: Pulmonary Vascular Resistance, HIV: Human Immunodeficiency Virus, BMI: Body Mass Index.

**Absolute Contraindications**
Age > 70 years
Severe pulmonary hypertension with PAS > 50 mm Hg, TPG > 15 mm Hg, PVR > 3 Wood Units irreversible with milrinone/levosimendan
Severe lung disease (e.g., forced expiratory volume in one second; and forced vital capacity <50% of predicted value) or evidence of parenchymal lung disease)
Multisystem disease with poor long-term survival
Viral infection (hepatitis B, hepatitis C, and HIV) with organ damage and with detectable viral titles
Severe local or systemic infection not caused by left ventricular assist device
Active smokers/substance abuse
History of cancer (multidisciplinary cardio-oncology team evaluation is recommended)
Severe neurological deficit/significant psychiatric illness
**Relative contraindications**
Diabetes mellitus with end-organ damage (e.g., nephropathy, neuropathy, proliferative retinopathy) or poorly controlled diabetes with glycosylated hemoglobin persistently >7.5% or 58 mmol/mol
Irreversible renal dysfunction with an estimated glomerular filtration rate <40 mL/min/1.73 m^2^ if the patient is not a candidate for a combined heart-kidney transplant
Irreversible liver dysfunction (e.g., cirrhosis) if the patient is not a candidate for a combined heart-liver transplant.
Severe obesity (BMI > 35 kg/m^2^)
Psychosocial factors (Inability to make a solid commitment to transplantation, Absence of adequate external psychosocial supports)

**Table 3:** Parameter for evaluation of kidney function in patients evaluated for heart transplantation. eGFR: estimated glomerular filtration rate. CKDEPI: Chronic Kidney Disease Epidemiology Collaboration, eGFR: estimated Glomerular Filtration Rate.

Parameter	Note
Intrinsic renal disease	The presence of hypertension, diabetes mellitus, or lupus increases the possibility of irreversible kidney disease.
eGFR	Perform two independent measurements for eGFR at least two weeks apart with the CKD-EPI formula.
Proteinuria	Proteinuria is indicative of intrinsic kidney damage.
Kidney size and parenchymal evaluation	The shrunken size of the kidney and the presence of cortical scarring indicate long-term intrinsic and irreversible kidney disease.
Improved kidney function after hemodynamic optimizations	Increased eGFR after the use of inotropes/vasodilators/diuretics is indicative of functional kidney dysfunction.

**Table 4:** Clinical marker presence required a hepatological evaluation in patients evaluated for heart transplantation. MELD-XI: Model for End-Stage Liver Disease Excluding INR.

**Marker of Advanced Liver Disease**
Nodular liver contour
Splenomegaly
Varices
**Marker of suspected liver disease**
Hypoalbuminemia
High International Normalize Ratio in patients not anticoagulated
Elevated bilirubin
MELD-XI scores > 11
Ascites
Thrombocytopenia

## Data Availability

No new data were created or analyzed in this study. Data sharing is not applicable to this article.
